# A longitudinal observational study of male referrals and adherence to imaging guidelines: a single centre experience in 148 patients

**DOI:** 10.3389/fonc.2026.1769477

**Published:** 2026-04-07

**Authors:** Olivia Pestrin, Joshua Agilinko, Syeda Hajra Basharat, Taiwo Ali, Tajudeen Wahab

**Affiliations:** 1 Elm Breast Care Centre, Barking Havering and Redbridge University Hospitals NHS Trust, Romford, United Kingdom; 2 Department of Surgery and Cancer, Imperial College, London, United Kingdom

**Keywords:** gynaecomastia, male breast disease, imaging guidelines, Association of Breast Surgery, diagnostic accuracy, retrospective study

## Abstract

**Introduction:**

Male breast cancer represents a rare clinical diagnosis, accounting for less than 1% of all breast cancers. The vast majority of male breast referrals are attributed to benign pathologies, most commonly gynaecomastia. Despite the low incidence of malignancy, variability in clinical acumen and a low threshold for concern frequently result in excessive imaging. To mitigate this, the Association of Breast Surgery (ABS) has issued national guidelines to standardise assessment and reduce unnecessary radiological exposure. This study aimed to evaluate male breast referrals to a high-volume UK breast unit over one year and assess adherence to ABS imaging recommendations.

**Methods:**

We conducted a retrospective analysis of all male patients referred to the symptomatic breast clinic at King George Hospital between January and December 2022. Clinical presentation, P-scores, radiological imaging (M and U scores), histopathological outcomes, and medication history were extracted from hospital records and multidisciplinary systems. Imaging pathways were critically appraised against 2021 ABS guidance, stratifying patients by age (<25 and ≥25 years).

**Results:**

A total of 148 male patients were assessed, with a mean age of 49.6 years (SD ± 22.3). The most frequent presentation was a unilateral breast lump (62.8%). Gynaecomastia was diagnosed in 97 patients (65.5%), with proton pump inhibitors implicated in over a quarter of these cases. Imaging was performed in 141 patients (95.3%), with 52.7% of all imaging deemed non-concordant with ABS guidance. Only 29.7% underwent imaging entirely in keeping with national recommendations. Eight patients (5.4%) were diagnosed with breast cancer, all over the age of 25. Notably, only one malignancy arose in a patient with a benign clinical examination (P1), flagged by indeterminate mammographic findings (M3). Advanced cross-sectional imaging (CT/MRI) was performed in 2% of the cohort.

**Conclusion:**

Despite clear national guidelines, our findings highlight a significant trend towards over-investigation in male breast referrals. The predominance of benign disease particularly drug-induced gynaecomastia underscores the need for judicious imaging. Adherence to ABS criteria must be reinforced, and closer collaboration between breast surgeons and radiologists is essential to optimise diagnostic yield, reduce patient anxiety, and conserve healthcare resources in the evaluation of the male breast.

## 1 Introduction

Male patients represent approximately 1% of referrals to symptomatic breast units in the United Kingdom (1). Pathologies of the male breast encompass both benign and malignant conditions, with gynaecomastia being the most frequently encountered benign diagnosis. Pseudo-gynaecomastia, a related but distinct entity caused by adipose tissue deposition without glandular proliferation, is commonly observed in individuals with elevated body mass index (2). The vast majority of cases of true gynaecomastia are benign in origin and can be safely managed in primary care without the need for specialist referral or imaging.

In contrast, male breast cancer remains a rare but clinically significant diagnosis, accounting for less than 1% of all breast cancers in the UK (3). Despite its rarity, outcomes in men tend to be inferior to those in women. Disease-specific mortality has been reported at 27.2% in males compared to 17.4% in females, likely reflecting a combination of delayed presentation, lower index of suspicion among both patients and clinicians, and the absence of routine screening in men (4).

Given the potential for life-limiting disease, distinguishing malignant from benign presentations in the male breast is of paramount importance. Accurate clinical assessment, supported by selective use of imaging and histological evaluation where appropriate, allows for timely cancer diagnosis while minimising unnecessary investigation in clearly benign cases.

A central challenge for breast cancer units is balancing the need for clinical vigilance with the appropriate use of finite radiological resources. The Association of Breast Surgery (ABS) provides clear national guidance on the indications for specialist referral and imaging in male breast disease (**Table 1**). For men under 25 years of age, imaging is generally not indicated unless the clinical examination suggests an indeterminate or suspicious mass (P3 or higher). In patients over 25 years, imaging is appropriate when there is clinical concern (P3–P5), typically with a dual-modality approach using ultrasound and mammography. Following imaging, a core biopsy is recommended for indeterminate (M3) or suspicious (M4/M5) radiological findings (5). Despite these well-defined parameters, variability in clinical practice persists. Numerous audits have highlighted a tendency toward over-investigation, including the use of imaging in clinically benign cases where guidelines would not support it (4, 6). This can lead to increased healthcare expenditure, unnecessary patient anxiety and excessive demand on radiological services. It is also important to recognise that radiological discretion plays a role in some centres, particularly in borderline clinical scenarios or when patient anxiety influences decision-making. While this flexibility allows for individualised care, it may also contribute to deviation from standardised pathways.

**Table 1:** The 2021 ABS guidelines for imaging in male patients (5).

Clinical scenario	Age < 25 years	Age ≥ 25 years
Bilateral gynaecomastia	No imaging	No imaging
Bilateral pseudogynaecomastia	No imaging	No imaging
Unilateral lump, clinically benign (P1–P2)	No imaging	No imaging
Unilateral lump, indeterminate (P3)	Ultrasound only	Ultrasound ± mammogram
Suspicious or malignant features (P4–P5)	Ultrasound ± core biopsy	Mammogram + ultrasound ± core biopsy
Indeterminate imaging findings (M3)	Core biopsy	Core biopsy

The aim of this longitudinal observational study was to evaluate male breast referrals to our unit over a 12-month period, with a specific focus on patterns of imaging use and adherence to ABS guidelines. Through this analysis, we aim to identify areas for improving compliance with national recommendations while ensuring safe and efficient management of male breast disease.

In the current NHS climate, the judicious use of diagnostic imaging has become increasingly important. Radiology services are under sustained pressure from rising referral volumes, workforce shortages, and expanding indications for imaging across multiple specialties. Unwarranted breast imaging in low-risk male patients contributes to service congestion, prolongs waiting times for higher-risk individuals, and exposes patients to unnecessary investigations and radiation. In parallel, there is growing recognition of the financial and environmental costs associated with avoidable imaging, particularly in publicly funded healthcare systems. A single centre study identifying inappropriate breast imaging resulting in additional requests accounting for €51,235 euro of unnecessary cost to a single centre in a 3-month period (7). Health system improvement analyses associated with guideline-driven best practice show substantial potential financial benefits. For example, the Get it Right the First Time (GIRFT) programme which promotes standardisation of care in alignment with ABS and other best-practice guidelines, identifies notional financial opportunities of £15m–.

£26m per year and estimated savings of £1.3m–£2.9m annually through increased day-case surgery and reduced bed-day use (8). This demonstrates that adhering to structured, guideline-based pathways in breast care can deliver measurable financial benefits to institutions. Ensuring adherence to evidence-based national guidance is therefore not only a matter of diagnostic accuracy, but also of healthcare sustainability, resource stewardship, and equitable access to specialist services.

## 2 Materials and methods

### 2.1 Study design and ethical considerations

This study was a retrospective observational evaluation conducted at the Barking, Havering and Redbridge University Hospitals NHS Foundation Trust. The project was registered and approved locally as a service evaluation through the Trust’s Quality and Safety Information System (QSIS). As the study utilised routinely collected, anonymised clinical data and did not involve any deviation from standard care pathways, formal review by a Research Ethics Committee was not required in accordance with UK Health Research Authority guidance. The study was conducted in line with institutional audit governance procedures and adhered to the principles of the Declaration of Helsinki.

### 2.2 Study setting and population

All male patients referred to the symptomatic one-stop breast clinic between 1 January and 31 December 2022 were included. The clinic operates within the NHS Two-Week Wait pathway for suspected breast cancer and provides integrated clinical assessment, imaging, and biopsy were indicated within a single visit (7).

Patients were stratified into two age groups: under 25 *and* 25 years or older. This stratification reflects the imaging thresholds outlined in the 2021 ABS guidelines for male breast disease, which recommends limited imaging in younger patients unless clinical concern is present (5). Patients with a prior diagnosis of primary or metastatic breast cancer were excluded to ensure the analysis focused on initial diagnostic pathways.

### 2.3 Clinical assessment

All patients underwent clinical breast examination by a consultant breast surgeon, specialist breast physician, or higher surgical trainee under consultant supervision. Clinical findings were recorded using the standardised UK five-point clinical scoring system: P1 (normal), P2 (benign findings including classical gynaecomastia), P3 (indeterminate), P4 (suspicious for malignancy), and P5 (clinically malignant). This scoring system facilitates structured risk stratification and informs subsequent decisions regarding imaging and biopsy.

### 2.4 Imaging and biopsy protocol

Imaging was performed according to clinical presentation, patient age, and national guidance. In men under 25 years of age, imaging was not routinely undertaken for clinically benign presentations (P1–P2), with ultrasound considered only in the presence of indeterminate or suspicious findings (P3 or higher). In men aged 25 years and over, imaging was performed when there was clinical concern (P3–P5), typically using mammography with or without adjunctive ultrasound.

Mammography was acquired in standard craniocaudal and mediolateral oblique views and reported using the Royal College of Radiologists five-point classification system (M1–M5). Ultrasound examinations were performed by consultant breast radiologists and graded using the corresponding U1–U5 scale.

Ultrasound-guided core needle biopsy was undertaken for indeterminate or suspicious imaging findings (M3/U3 or higher) or when there was discordance between clinical and radiological assessment. Core biopsies were performed using 14-gauge needles under local anaesthesia in accordance with unit protocol. Cross-sectional imaging with CT or MRI was arranged selectively where additional anatomical definition or staging was required, at the discretion of the multidisciplinary team. All patients undergoing biopsy were reviewed at the weekly breast multidisciplinary team meeting, involving surgery, radiology, pathology, oncology, and breast care nursing.

### 2.5 Data collection and management

Data were independently extracted by three authors from electronic health records and the institutional picture archiving and communication system (PACS). Variables collected included patient age, presenting symptoms, clinical examination score, imaging modality and radiological scores (M and U), biopsy status, histopathological findings, and final clinical outcome. Any discrepancies in data extraction were resolved by consensus, with input from a fourth senior author where required.

All data were anonymised prior to analysis and stored in a secure, password-protected Microsoft Excel spreadsheet (version 2304), accessible only to members of the study team.

### 2.6 Statistical analysis

Statistical analysis was performed using MedCalc Software Ltd (version 22.009) (9). Descriptive statistics were used to summarise patient demographics, clinical presentation, imaging utilisation, and histopathological outcomes. Categorical variables are reported as absolute numbers and percentages. Continuous variables are presented as means with standard deviations, where appropriate, to describe the overall referral population.

In addition to descriptive analysis, exploratory inferential statistics were performed to contextualise cancer detection patterns. Comparisons between categorical variables were assessed using Fisher’s exact test (10). Fisher’s exact test was selected for analysis of categorical variables due to the small sample sizes and low event rates observed in several comparison groups, particularly for malignant outcomes. Cancer detection rates are reported with 95% confidence intervals calculated using exact binomial methods to reflect uncertainty around rare outcomes, including malignancy in clinically benign presentations. Statistical significance was defined as a two-sided *p* value < 0.05.

## 3 Results

### 3.1 Patient demographics and referral indications

A total of 148 male patients were reviewed at the King George Breast Unit between 5 January and 31 December 2022. The mean age at presentation was 49.6 years (SD. ± 22.3).

The most common reason for referral was a unilateral breast lump (n = 93, 62.8%), followed by breast enlargement (n = 17, 11.5%) and mastalgia (n = 15, 10.1%). Bilateral breast lumps, nipple changes, and incidental findings accounted for smaller proportions.

### 3.2 Imaging utilisation

Of the 148 patients, 141 (95.3%) underwent radiological imaging. In seven cases, benign clinical assessment (P1–P2) did not warrant imaging and no further investigation was performed. Among those imaged, 52 patients (36.9%) underwent ultrasound alone, four (2.8%) had mammography alone, and 70 (49.6%) received both ultrasound and mammography. Eleven patients (7.8%) underwent dual imaging followed by core biopsy. Overall, 89 of 148 patients (60.1%) underwent mammography as part of their diagnostic workup. A further four patients (2.0%) underwent additional cross-sectional imaging with CT or MRI. These imaging patterns are summarised in **Table 2**.

**Table 2:** Modalities of clinical imaging utilised to assess 141 male patients.

Modality of imaging	Number of patients	Percentage of patients (%)
US only	52	36.9
Mammogram only	4	2.8
US and mammogram	70	49.6
US, mammogram, and core biopsy	11	7.8
US, mammogram and Computer Tomography/Magnetic Resonance Imaging	4	2

### 3.3 Adherence to ABS imaging guidelines

Patients were stratified by age: 33 (22.3%) were under 25 years, and 115 (77.7%) were aged 25 years or older. In the under-25 group, 28 patients underwent ultrasound alone, two received dual-modality imaging, one had mammography alone, and two did not undergo any imaging. According to 2021 ABS guidance, imaging is not routinely indicated in patients under 25 years with clinically benign findings (P1–P2). Applying this criterion, five patients received guideline-concordant management (three appropriately imaged and two appropriately not imaged), while 28 patients (84.8%) underwent imaging outside guideline recommendations. No malignancies were diagnosed in this age group. In the ≥25-year cohort, all 115 patients underwent imaging. Based on ABS guidance recommending imaging for P3 or higher clinical findings, 55 patients were appropriately investigated. The remaining 60 patients (52.2%) were over-investigated relative to guideline recommendations; all had a benign clinical examination (P1). Among these patients, 42 underwent dual-modality imaging, 14 had ultrasound alone, and four received mammography alone. One patient in this group had an indeterminate mammographic finding (M3) and was subsequently diagnosed with invasive ductal carcinoma. This represents the only malignancy detected among patients with a clinically benign presentation.

### 3.4 Correlation between clinical assessment, imaging and histology

Eight of the 148 male patients included in the study were diagnosed with breast cancer, corresponding to an overall malignancy rate of 5.4% (95% CI approximately 2.4–10.3%). All malignancies occurred in patients aged 25 years or older. Seven of the eight patients (87.5%) had a clinically indeterminate or suspicious examination (P3 or higher) at presentation. One patient had a clinically benign examination (P1) but was escalated appropriately following an indeterminate mammographic finding (M3) and was diagnosed with invasive breast cancer on core biopsy.

Malignancy was significantly more common in patients with clinically suspicious examinations (P3–P5) compared with those with benign findings (P1–P2) (Fisher’s exact test, p < 0.01). Among patients with a P1 examination, the cancer detection rate was low, corresponding to approximately 1–2%, reflecting the rarity of malignancy in this group.

Biopsies were otherwise performed in patients with indeterminate or suspicious clinical and/or imaging findings (P3+, M3+, or U3+), in accordance with national protocols.

### 3.5 Benign and malignant diagnoses

**Table 3** summarises final diagnoses across the cohort. The most common diagnosis was gynaecomastia, identified in 97 patients (65.5%). Other benign diagnoses included normal breast tissue (n = 24, 16.2%), sebaceous cysts (n = 13, 8.8%), and lipomas (n = 7, 4.7%). Only one of the eight patients diagnosed with breast cancer had a clinical score less than P3, corresponding to 0.68% of the total cohort.

**Table 3:** Diagnosis of male patients presenting to breast clinic.

Clinical diagnosis	Number of patients	Percentage (%)
Gynaecomastia	97	65.5
Normal breast tissue	24	16.2
Sebaceous cyst	13	8.8
Lipoma	7	4.7
Breast cancer	8	5.4

#### 3.5.1 Patterns of gynaecomastia

Among the 97 patients diagnosed with gynaecomastia, 79 (81.4%) had bilateral disease (Graph 2), with asymmetry noted in nine cases. Unilateral gynaecomastia was observed in 16 patients, involving the right breast in nine and the left in seven cases. Two patients were diagnosed with pseudo-gynaecomastia, characterised by subcutaneous adipose tissue without glandular proliferation.

### 3.6 Clinicopathological features of breast cancer

The clinicopathological characteristics of the eight patients diagnosed with breast cancer are detailed in **Table 4**. Patient ages ranged from 41 to 85 years. Most.

**Table 4:** Associated characteristics of breast cancer associated in 8 patients.

Patient	Age	Reason for referral	Clinical exam score	Mammo gram score	USS score	Tumour type	Tumour size on histology	Grade	Tumour markers	Axillary status *(on imaging)*	Distant mets *(on imaging)*
1	79	Breast Lump	P5	5	5	Infiltrating Ductal Carcinoma	18 mm	1	Nil	Prominent Lymph Node	Nil
2	68	Incidental right breast lesion on PET for prostate cancer	Unknown	5	5	invasive ductal carcinoma	15 mm	2	ER	Clear	Co-commitant Prostate cancer nil mets
3	85	Axillary skin rash	P5	2	2	invasive carcinoma unknown origin	Not excised	N/A	ER	Clear	N/A
4	47	2 yr history of axillary lymph node increasing in size	P1	3	1	unknown	37 mm	1	HER2	+ node	Nil
5	41	Breast Lump	P3	5	5	invasive ductal carcinoma	25 mm	3	ER	Clear	Nil
6	82	Breast Lump	P5	5	5	invasive ductal carcinoma	Not excised	3	ER	Clear	Peripheral centrilobular nodules: probable lymphangitis carcinomatosis
7	80	Lateral Upper border axillary lump	P5	3	5	Invasive ductal carcinoma	Not excised	4	HER2	N/A	Co-committant lung cancer
8	64	Incidental finding of breast lesion on CT scan	P4	5	5	tubular invasive ductal carcinoma	45 mm	2	ER	Clear	Nil

presented with a palpable breast lump and had P3–P5 clinical scores. Histological subtypes included invasive ductal carcinoma and tubular carcinoma. Two malignancies were diagnosed incidentally during imaging for other primary cancers (prostate and lung). Distant metastases were identified in one patient with lymphangitic spread on CT imaging, and one patient had synchronous lung cancer.

#### 3.6.1 Medications associated with gynaecomastia

Among patients diagnosed with gynaecomastia, medication history was contributory in a substantial proportion (**Table 5**). Proton pump inhibitors were the most frequently recorded drug (n = 27, 27.8%), followed by calcium channel blockers (n = 16, 16.5%) and statins (n = 14, 14.4%). Additional medications included spironolactone, anabolic steroids, and antipsychotic agents. Eleven patients (11.3%) were classified as having polypharmacy.

**Table 5:** Patients diagnosed with gynecomastia utilizing drugs associated with the development of gynecomastia.

Medications	Number of patients	Percentage
None	37	38.1
Not Documented	17	17.5
PPI	27	27.8
CCB	16	16.5
Statins	14	14.4
Recreational Drugs	8	8.2
Spironolactone	5	5.2
Alpha-blocker	5	5.2
Anabolic Steroids	3	3.1
Anti-Psychotics	2	2.1
Analgesics	2	2.1
Anti-androgen	1	1.0
Polypharmacy	11	11.3

## 4 Discussion

### 4.1 Summary of findings

In this retrospective observational study, we evaluated all male patients referred to a single tertiary breast unit over a 12-month period to assess imaging utilisation and adherence to national guidance. The cohort was predominantly middle-aged, with unilateral breast lump the most common presenting symptom. Imaging was performed in the vast majority of patients, frequently using dual-modality approaches, despite a high prevalence of benign disease.

Gynaecomastia was the most frequent diagnosis, accounting for almost two-thirds of cases, followed by normal breast tissue and other benign lesions. Breast cancer was diagnosed in eight patients (5.4%), although two cases were detected incidentally during investigations for other primary malignancies. Excluding incidental findings, the primary cancer detection rate among symptomatic referrals was low. This reflects the identified wider rate of Male breast cancer accounting for less than 1% of all breast cancers in the UK, and median diagnostic age reflecting the established increased incidence with age life (3, 11). Importantly, nearly all malignancies occurred in patients with clinically indeterminate or suspicious examinations (P3 or greater).

### 4.2 Adherence to ABS imaging guidance and clinical safety

The Association of Breast Surgery (ABS) provides national guidance for the assessment and imaging of male breast disease, aiming to balance early cancer detection with avoidance of unnecessary investigation (5). These recommendations prioritise clinical examination, reserving imaging for indeterminate or suspicious presentations and applying age-specific thresholds to guide modality selection. When benchmarked against this framework, our findings demonstrate a substantial degree of imaging outside guideline recommendations (88/148), particularly among patients with clinically benign examinations, despite the very low prevalence of malignancy in this group. Identifying that patients under the age of 25 (28/33) having higher rates of guidance discordance than those >25 (60/116).

Similar deviations from guideline-based pathways have been reported in previous audits and likely reflect a combination of diagnostic caution, ease of access to imaging, and variability in decision-making within multidisciplinary breast services (12). In the contemporary NHS setting, breast assessment is delivered by an increasingly diverse clinical workforce, including surgeons, breast physicians, and advanced practitioners, which may contribute to heterogeneity in guideline interpretation. However, although most male breast referrals arise from general practice via the Two-Week Wait pathway, referrer-level data were not consistently available in this study. Consequently, it was not possible to determine whether imaging utilisation or guideline adherence varied by referral source, and observed patterns should be interpreted as reflecting downstream diagnostic decision-making within secondary care rather than referral behaviour alone. One malignancy in this cohort occurred in a patient with a clinically benign examination (P1) and was identified following indeterminate mammographic findings. This case highlights the inherent tension between diagnostic efficiency and patient safety when applying guideline-based imaging pathways. While strict adherence to ABS guidance would have reduced imaging in a large proportion of clinically benign cases, this example underscores that guidelines are intended to support, rather than replace, clinical judgement. Notably, this represented a very small proportion of the cohort and does not undermine the overall sensitivity of clinical assessment with literature citing clinical examination as 99.4% negative predictive value (NPV) for male breast cancer diagnosis, and vast majority of malignancies occurred in patients with P3 or higher examinations. In the absence of long-term follow-up, conclusions regarding diagnostic safety should be interpreted in the context of short-term outcomes (13).

### 4.3 Imaging modality and diagnostic yield

Ultrasound was the most frequently used imaging modality in this cohort, whereas mammography alone was uncommon. Current ABS guidance recommends ultrasound as first-line imaging in men under 25 years of age and mammography with or without ultrasound in older patients when clinical concern exists. Published literature has demonstrated high diagnostic accuracy for mammography in male breast disease, with superior specificity for distinguishing benign from malignant lesions in several large series (13–15). Mammography may also offer advantages in characterising benign entities such as sebaceous cysts and calcified lesions.

However, this study was not designed to directly compare the diagnostic performance of imaging modalities within its own cohort. As such, inferences regarding relative accuracy must be interpreted in the context of existing literature rather than derived from the present data. Mammography may therefore represent a valuable component of the diagnostic pathway in selected male patients over 25 years of age, but the optimal sequencing and prioritisation of imaging modalities remains an open question and warrants prospective evaluation rather than immediate modification of current guideline recommendations.

### 4.4 Gynaecomastia and medication use

Almost half of patients diagnosed with gynaecomastia were taking medications previously associated with its development, most commonly proton pump inhibitors, calcium channel blockers, and statins. Although these agents are widely prescribed in the general population, the proportion observed in this cohort suggests that medication-related gynaecomastia may be an under-recognised contributor to male breast referrals. Documentation of medication review or discontinuation prior to referral was inconsistent, despite ABS recommendations that potentially causative drugs should be stopped or substituted where possible (16, 17).

The ABS also advises that primary care clinicians undertake relevant endocrine and systemic investigations before referral, including hormonal profiles and liver and renal function tests (18). We were unable to verify whether such investigations were routinely performed. Although longitudinal data were not available to assess symptom resolution following medication withdrawal, these findings highlight an opportunity to strengthen primary care assessment, which may reduce unnecessary specialist referral and imaging in patients with clinically benign gynaecomastia.

### 4.5 Implications for clinical practice and policy

This study demonstrates persistent over-utilisation of breast imaging in men with low-risk clinical presentations, in keeping with prior evidence (19, 20). While imaging is essential when clinical uncertainty exists, suboptimal adherence to national guidance contributes to unnecessary investigations, patient anxiety, and avoidable pressure on radiology services.

Although this study did not include a formal cost-effectiveness or radiation exposure analysis, the observed volume of imaging outside guideline recommendations has clear resource implications. Using conservative NHS reference cost estimates, potentially avoidable imaging in this cohort would equate to several thousand pounds in diagnostic expenditure over a 12-month period at a single centre. Based on the observed non-compliance with Association of Breast Surgery (ABS) guidelines and the NHS-reported tariffs for mammography (£64.62) and ultrasound (£32.82), the centre may have accrued an estimated excess cost of ~£5,730 (21). This reflects the additional expenditure associated with imaging performed outside guideline recommendations, highlighting the financial impact of unnecessary investigations and the potential savings achievable through stricter adherence to ABS best-practice standards. Another, single centre study identifying inappropriate breast imaging resulted in an additional cost of 51,235 euro in a 3-month period (7). Beyond direct costs, unnecessary imaging increases radiology workload and may delay access for higher-risk patients.

Strengthening primary care assessment, reinforcing guideline-based pathways, and improving multidisciplinary collaboration between surgeons, radiologists, and referrers are likely to enhance diagnostic efficiency while maintaining patient safety. Reflecting on the benefits demonstrated by the GIRFT programme, future prospective studies assessing and implementation of ABS guidelines incorporating long-term follow-up and health-economic evaluation may further refine imaging thresholds in male breast disease; contributing to standardization of patient care. Improved guideline alignment has the potential to reduce unwarranted variation in clinical practice, optimise the use of diagnostic imaging, and minimise unnecessary investigations (8, 22). Collectively, these improvements may contribute to more efficient use of healthcare resources and a reduction in overall health system costs, while maintaining high standards of diagnostic accuracy and patient safety.

## 5 Conclusion

Gynaecomastia accounted for the majority of male breast referrals in this cohort, while malignancy was uncommon and predominantly associated with clinically suspicious examinations. Despite clear national guidance, fifty nine percent of reviewed patients underwent imaging outside ABS recommendations, most often in clinically benign scenarios. Improved adherence to risk-stratified imaging pathways has the potential to reduce unnecessary investigations and pressure on radiology services without compromising short-term cancer detection. While mammography may offer diagnostic advantages in selected patients, imaging decisions should remain guided by clinical assessment and national recommendations. Enhancing primary care evaluation and multidisciplinary collaboration may reduce variation in practice and optimise management of male breast disease.

## 6 Strengths and limitations

This study includes a complete dataset of all male patients referred to a high-volume breast unit over a 12-month period, providing a robust snapshot of real-world diagnostic practice. The use of standardised clinical and radiological scoring systems enabled consistent assessment of imaging utilisation and adherence to ABS guidance. Limitations include the single-centre, retrospective design, which may limit generalisability. The retrospective design is inherently subject to selection and information bias due to reliance on existing clinical records. Documentation completeness and accuracy could not be controlled, and missing data — particularly regarding medication history and primary care investigations — may have influenced the assessment of referral appropriateness and diagnostic pathways, prevents assessment of referral behaviour. Future studies reviewing rates of referral from providers as well as investigations/interventions trialled prior to referral in future study would be of benefit. Future research should incorporate inferential statistical analyses in a multicentre study to strengthen generalisability, and direct future policy change. Formal cost and radiation dose analyses were not performed, and cost estimates are illustrative. Finally, the analysis focused on outcomes at initial presentation, and long-term follow-up was not available to identify delayed diagnoses. Consequently, conclusions regarding diagnostic safety and negative predictive value are based on short-term outcomes and should be interpreted with appropriate caution.

## Data Availability

The raw data supporting the conclusions of this article will be made available by the authors, without undue reservation.
